# Vitamin D deficiency and asthma morbidity in school-age children: a single-center cohort study

**DOI:** 10.3389/fpubh.2025.1717912

**Published:** 2025-12-15

**Authors:** Lijuan Dong, Yan Li

**Affiliations:** Department of Pediatrics, The Second Affiliated Hospital of Xi'an Jiaotong University, Xi'an, Shaanxi, China

**Keywords:** pediatric asthma, vitamin D deficiency, asthma control, exacerbation frequency, inhaled corticosteroids, environmental risk factors

## Abstract

**Background:**

Asthma remains a major cause of morbidity among school-age children worldwide, with deficiency of vitamin D increasingly recognized as a modifiable factor influencing immune regulation and respiratory health. However, evidence on its association with asthma control and exacerbation patterns in pediatric populations is limited, in low- and middle-income population.

**Methods:**

A retrospective single-center study of 320 children, 6–14 years, diagnosed with asthma between January 2019 and December 2023. Following application of inclusion and exclusion criteria, 210 children were analyzed. Data were obtained from medical records and structured questionnaires, including socio-demographic characteristics, asthma control status, exacerbation frequency, hospitalization history, and biochemical assays (serum vitamin D, calcium, phosphate, parathyroid hormone). Patients were categorized as vitamin D deficient, insufficient, or sufficient. Statistical analyses, including χ^2^ tests, ANOVA, and multivariable logistic regression, were performed using R (v4.3.2).

**Results:**

Vitamin D deficiency was prevalent in 45.2% of children, while 31.0% were insufficient and 23.8% sufficient. Poor asthma control was more common among deficient children (63.2%) compared to insufficient (43.1%) and sufficient groups (24.0%). The mean annual exacerbation rate was highest in the deficient group (2.6 ± 1.3) vs. insufficient (1.9 ± 1.1) and sufficient groups (1.1 ± 0.9). Hospitalization rates also differed significantly (31.6, 18.5, and 12.0%, respectively; *p* = 0.014). Multivariable logistic regression confirmed that vitamin D deficiency independently predicted poor asthma control (adjusted OR: 2.8, 95% CI: 1.6–4.9, *p* < 0.001) after adjustment for age, sex, BMI, and treatment profile.

**Conclusion:**

Vitamin D deficiency is highly prevalent among school-age children with asthma and is independently associated with poor control, higher exacerbation frequency, and increased hospitalizations. As part of integrated pediatric asthma care, these findings emphasize the significance of routine screening and the need to take vitamin D supplements into account. At the public health level, targeted nutritional interventions may reduce disease burden and improve long-term outcomes in children.

## 1 Introduction

One of the most common chronic respiratory conditions affecting school-age children, asthma has a significant negative influence on quality of life, academic performance, and healthcare use, making it a significant public health burden (1, 2). Characterized by recurrent wheezing, airway inflammation, and variable airflow limitation, asthma in children often remains poorly controlled, leading to frequent exacerbations and hospitalizations (3). These outcomes highlight not only the clinical but also the societal costs of uncontrolled asthma, reinforcing the need for preventive strategies that target modifiable risk factors (3).

Among potential determinants, vitamin D deficiency has gained attention for its immunomodulatory role in respiratory health (4). Vitamin D influences immune balance, regulates inflammatory pathways, and supports antimicrobial defenses, mechanisms that are highly relevant in the context of airway inflammation and asthma exacerbations (5). Deficient levels have been linked with increased susceptibility to respiratory infections and more severe asthma manifestations (6, 7). Vitamin D status monitoring could be a useful way to identify kids who are at risk of uncontrolled asthma and its associated problems (8).

Evidence from clinical studies indicates that vitamin D supplementation can reduce exacerbation frequency and improve lung function in pediatric asthma, while observational research supports a relationship between deficiency and poorer outcomes (9). However, knowledge gaps remain regarding how these associations translate into real-world patient populations (7). Many existing studies have focused on intervention trials with selective inclusion criteria, leaving limited evidence from hospital-based cohorts where children present with diverse socio-demographic and clinical profiles (10). Addressing this gap requires systematic analysis of routine patient data, which can provide insights into population-level patterns and disparities relevant to public health practice (11).

This study was conducted to evaluate the association between vitamin D deficiency, asthma control, and exacerbation frequency in school-age children using retrospective hospital data. Sensitivity analyses using alternative cutoff values and subgroup analyses by age and sex were also performed. This study offers fresh proof of vitamin D's function as a modifiable predictor of pediatric asthma by fusing a clinical outcome framework with a public health viewpoint. It is anticipated that the results will help achieve Sustainable Development Goal 3, which is to ensure children's health and wellbeing, and will guide community-based prevention measures.

## 2 Methods

### 2.1 Study design

The study was conducted at the Pediatric Department of The Second Affiliated Hospital of Xi'an Jiaotong University, a tertiary care referral center in Xi'an, Shaanxi, China. Data collection was carried out between 2019 and 2023. The study population consisted of children aged 6–14 years with a confirmed physician diagnosis of asthma following the Global Initiative for Asthma (GINA) guidelines (2). The diagnosis of asthma was confirmed based on established clinical criteria, which included the presence of recurrent wheezing, physician assessment, and documented responsiveness to bronchodilator therapy. Indications for hospitalization were defined as acute exacerbations requiring medical observation and intervention. The duration of asthma diagnosis and controller (e.g., inhaled corticosteroids, long-acting beta-agonists, leukotriene receptor antagonists) and reliever (e.g., short-acting beta-agonists) medication use was recorded from medical records, in accordance with recommended continuous controller therapy for at least 6 months and reliever use as needed for symptom control, as per international asthma management guidelines Global Initiative for Asthma (GINA); Global Strategy for Asthma Management and Prevention, 2025 (12). Ethical approval was obtained from the Institutional Review Board of The Second Affiliated Hospital of Xi'an Jiaotong University (No. 2024034). Informed permission was not required because the study was anonymized and retrospective in terms of record review.

### 2.2 Participants

**Figure 1:**
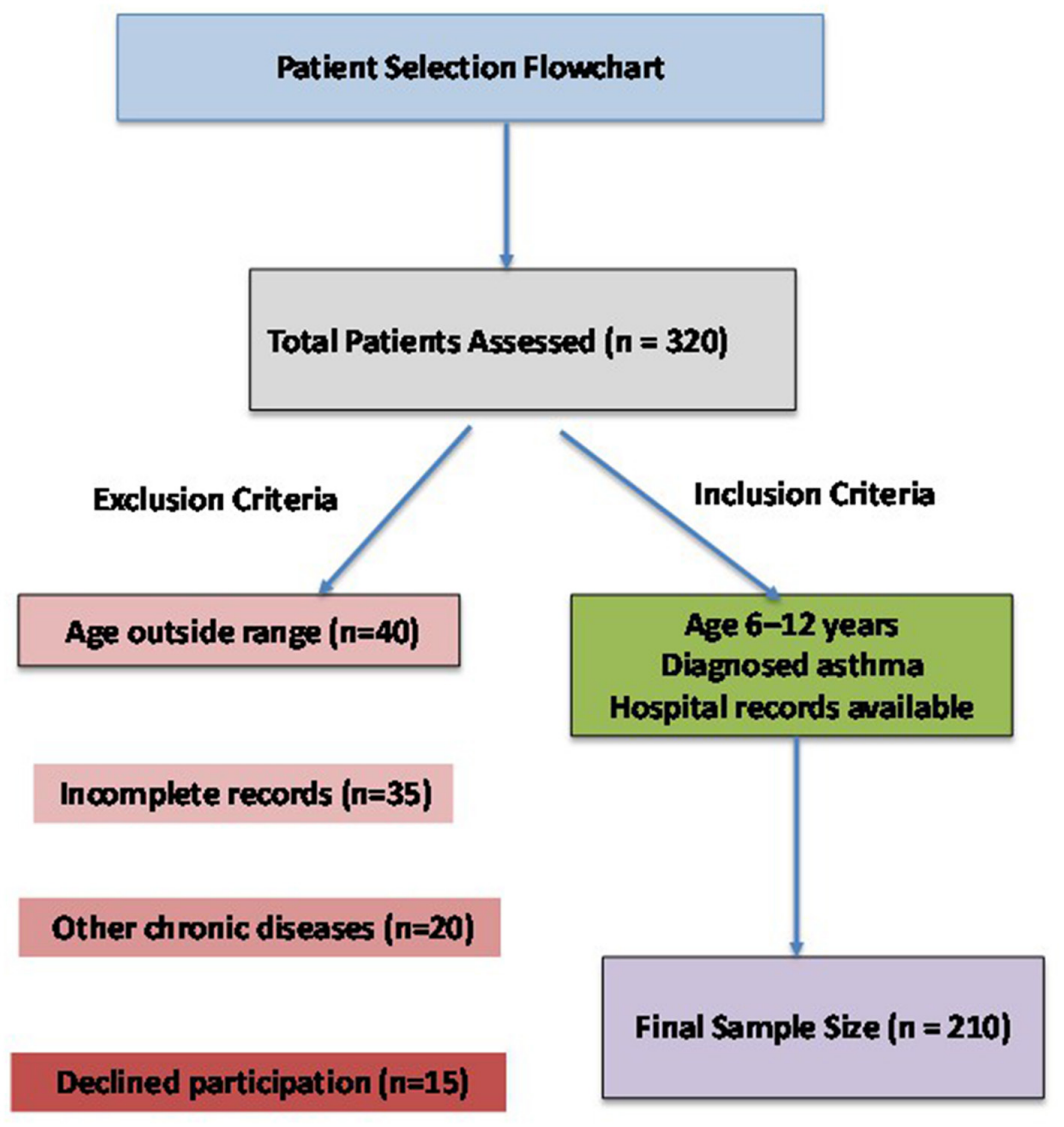
Flow chart illustrating the selection of participant selection for retrospective cohort study.

Eligible participants were children diagnosed with asthma who attended routine outpatient visits or were admitted for follow-up care during the study period. Exclusion criteria included children with chronic pulmonary disorders other than asthma, congenital anomalies, or incomplete medical records (13, 14). Demographic details (age, sex, weight, height, body mass index), family history, and duration of asthma were retrieved from medical charts. Caregivers were invited to complete a structured questionnaire to assess exposure, lifestyle, and adherence patterns related to asthma management (4). Figure 1 is showing the selection of participant selection for retrospective cohort study.

### 2.3 Questionnaire development

This study was conducted as a retrospective analysis of children with physician-diagnosed asthma according to GINA guidelines; hence, a healthy control group was not included. Relevant clinical data, including asthma duration, reliever medication use, exacerbation frequency, hospitalization history, and prior treatments, were carefully extracted from medical records to provide context for asthma control. A structured questionnaire (Supplementary Table S1) was developed to capture four domains relevant to childhood asthma and vitamin D status: (1) sunlight exposure, (2) nutrition and supplementation, (3) environmental risk factors, and (4) healthcare access and adherence (2, 4). Items were adapted from validated surveys used in pediatric asthma and public health studies, with modifications for local cultural context. Responses were recorded on categorical scales to allow quantification into composite scores.

### 2.4 Scoring framework

Each domain was operationalized into a composite score (Supplementary Table S2). The Sunlight Exposure Score (SSES, range 0–5) reflected outdoor playtime, clothing coverage, and sunblock use (15). The Nutrition and Supplementation Score (NSS, range 0–6) measured daily milk intake, fish or egg consumption, and vitamin D supplementation (16). The Environmental Risk Score (ERS, range 0–3) assessed exposure to passive smoking, biomass fuel, and household crowding. The Healthcare Access and Adherence Score (HAAS, range 0–3) evaluated follow-up visits, controller medication use, and reported adherence. Higher scores indicated greater protective behaviors in SSES, NSS, and HAAS, while higher ERS scores reflected greater environmental risk (3, 4, 17).

### 2.5 Outcome measures

Asthma outcomes comprised the number of exacerbations in the preceding 12 months, frequency of symptoms, night-time awakenings, use of reliever medications, and history of hospitalizations. Lifestyle factors, such as sunlight exposure and dietary vitamin D intake, were also documented (5). The level of Vitamin D was categorized as deficient, insufficient, or sufficient. Asthma control was categorized as “controlled” or “poorly controlled” according to GINA criteria, which consider symptom frequency, reliever use, and night-time awakenings over the past 4 weeks (4, 18).

### 2.6 Statistical analysis

Data were analyzed using SPSS version 26 (IBM Corp., Armonk, NY) and R version. Continuous variables were presented as mean ± standard deviation, and categorical variables as frequencies and percentages (5). Associations between domain scores and asthma outcomes were examined using logistic regression for binary outcomes (control status, hospitalization), Poisson or negative binomial regression for count data (exacerbations), and linear regression for continuous severity indices. Multivariable models adjusted for age, sex, and body mass index. Statistical significance was defined at *p* < 0.05 (19).

### 2.7 Public health relevance statement

This study addresses an important intersection of pediatric asthma and nutritional deficiency within a public health framework. Vitamin D deficiency has emerged as a modifiable risk factor for poor asthma outcomes, yet its contribution in real-world community settings remains underexplored (19, 20). By integrating biochemical parameters with lifestyle and environmental risk factors, this research provides actionable insights into preventable determinants of asthma control. The findings support population-based strategies such as dietary interventions, outdoor activity promotion, and environmental exposure reduction. These results align with Sustainable Development Goal 3, “Good Health and WellBeing,” by advancing understanding of preventable childhood morbidity (19, 20).

## 3 Results

### 3.1 Baseline characteristics of the study population

**Figure 2:**
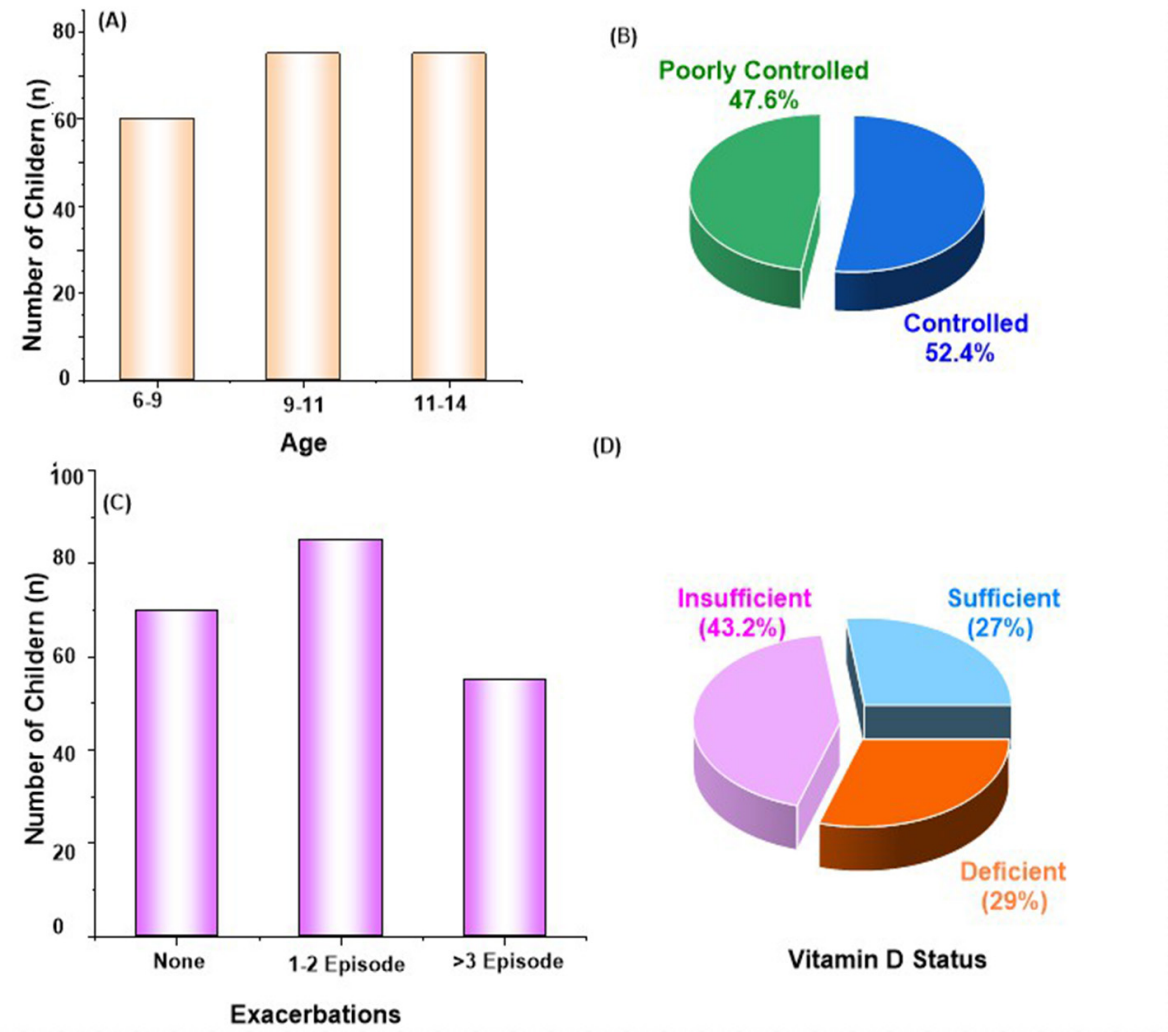
Socio-demographic and clinical characteristics of children; **(A)** age, **(B)** asthma control, **(C)** exacerbations in past 12 months, **(D)** vitamin D status.

Among 210 children with asthma, the mean age was 9.4 ± 2.1 years, with a slightly higher proportion of males (55.2%) which aligns with prior epidemiological evidence indicating higher asthma prevalence in boys during childhood (Figure 2). The mean BMI was 18.1 ± 3.2 kg/m^2^, with 22.4% of children classified as overweight or obese (3). A positive family history of asthma was reported in 39.5% of participants. Socioeconomic distribution showed that 47.6% belonged to middle-income households. It is evident with a larger proportion of children from lower-income families presenting with vitamin D deficiency, consistent with global trends linking nutritional limitations to disease burden (Supplementary Table S3). Questionnaire responses revealed that 63.2% of caregivers had limited awareness of asthma triggers, and 58.1% reported irregular use of prescribed medications. Sunlight exposure <30 min daily was reported in 61.0% of the children (15). These findings underscore the relevance of social determinants of health in asthma control within pediatric populations.

### 3.2 Biochemical parameters and treatment profile

The mean serum vitamin D level was 19.2 ± 6.1 ng/ml, with 68.6% of children below the recommended sufficiency threshold. Serum calcium remained largely within the normal range (8.7 ± 0.9 mg/dl). Nearly half of the children (46.7%) were receiving vitamin D supplementation, yet deficiency persisted in a majority. Among treatments, 57.1% reported regular inhaled corticosteroid (ICS) use, 78.6% used short-acting β_2_-agonists (SABA), and 22.9% required systemic steroids within the past year. Hospitalization due to asthma exacerbation was reported in 22.9% of participants. Additional biochemical distributions are detailed in Supplementary Table S4, while treatment breakdowns are expanded in Supplementary Table S5. Together, these tables provide a broader context to the physiological and therapeutic landscape of asthma management in children.

### 3.3 Vitamin D status and asthma outcomes

Unadjusted analysis demonstrated significant differences in asthma outcomes across vitamin D status categories. Poorly controlled asthma was most frequent among deficient children, with 60 of 95 (63.2%) affected, compared with 28 of 65 (43.1%) in the insufficient group and 12 of 50 (24.0%) in the sufficient group. Mean exacerbations in the past 12 months were highest in the deficient group (2.6 ± 1.3), intermediate in the insufficient group (1.9 ± 1.1), and lowest in the sufficient group (1.1 ± 0.9), with a significant overall difference (*F* = 32.1). Hospitalization rates followed a similar pattern: 30 of 95 (31.6%) among deficient children, 12 of 65 (18.5%) among insufficient, and 6 of 50 (12.0%) among sufficient (χ^2^ = 8.6). Sensitivity analyses using alternative vitamin D cutoffs (<12, 12–20, 20–30, >30 ng/ml) confirmed these trends (Supplementary Table S6), supporting the robustness of the findings.

**Figure 3:**
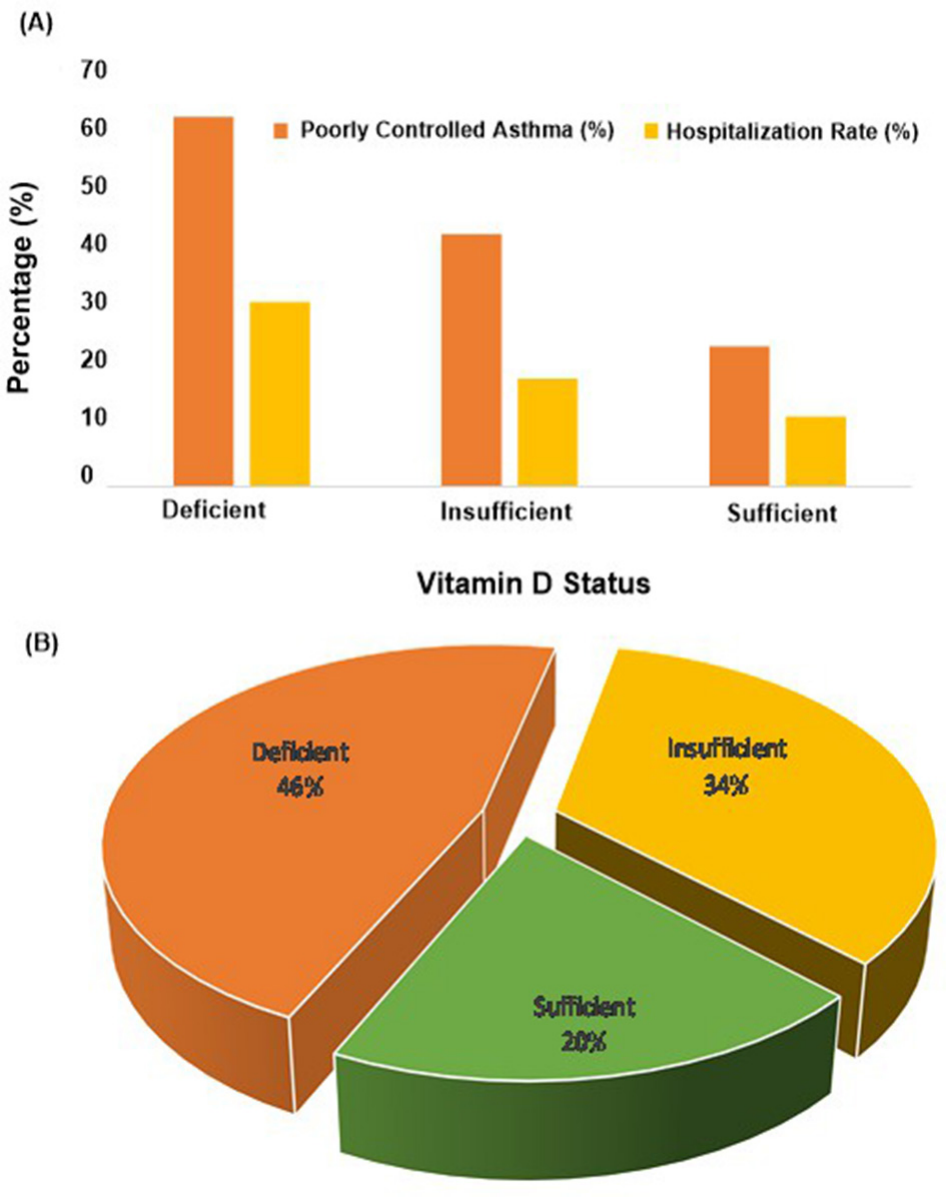
**(A)** Proportion of poor asthma control and hospitalizations by vitamin D status, **(B)** mean exacerbations in 12 months by vitamin D status.

Figure 3A shows that the proportion of poorly controlled asthma and hospitalization rates decreased progressively from vitamin D deficient to sufficient groups, highlighting a clear gradient in outcomes. Figure 3B demonstrates that children with deficiency experienced the highest mean number of exacerbations, whereas sufficient children had the lowest. Together, the figures emphasize the association between low vitamin D levels and worse asthma control, higher exacerbation burden, and greater healthcare utilization.

### 3.4 Statistical analysis

The logistic regression analysis identified the deficiency of vitamin D as a strong and independent predictor of poor asthma outcomes. Children with serum vitamin D levels below 20 ng/ml had more than twice the odds of poor asthma control (AOR: 2.38) compared with sufficient peers. Low outdoor activity (<1 h/day) was also associated with poor control (AOR: 1.95), while age, sex, and BMI showed no significant associations. For exacerbations, vitamin D deficiency was again a significant predictor, increasing the odds almost threefold (AOR: 2.71). Systemic corticosteroid use in the past year was the strongest predictor (AOR: 3.12), followed by passive smoke exposure (AOR: 1.89). Male sex was not associated with exacerbation risk (*p* = 0.850).

### 3.5 Multivariable logistic regression

The multivariable logistic regression model predicting poor asthma control in 210 children demonstrated that vitamin D deficiency (<20 ng/ml) was strongly associated with higher odds of poor control, with an adjusted odds ratio (OR) of 2.8 (95% CI: 1.6–4.9, *p* < 0.001). Passive smoking exposure also showed a significant association, increasing the risk nearly two-fold (OR: 1.9, 95% CI: 1.0–3.6, *p* = 0.04). Poor treatment adherence was another strong predictor with an OR of 2.6 (95% CI: 1.4–5.1, *p* = 0.003). In contrast, age, male sex, and BMI were not statistically significant, with ORs close to 1.0 and *p*-values above 0.05, suggesting these factors were not meaningful predictors of poor asthma control in this cohort.

**Figure 4:**
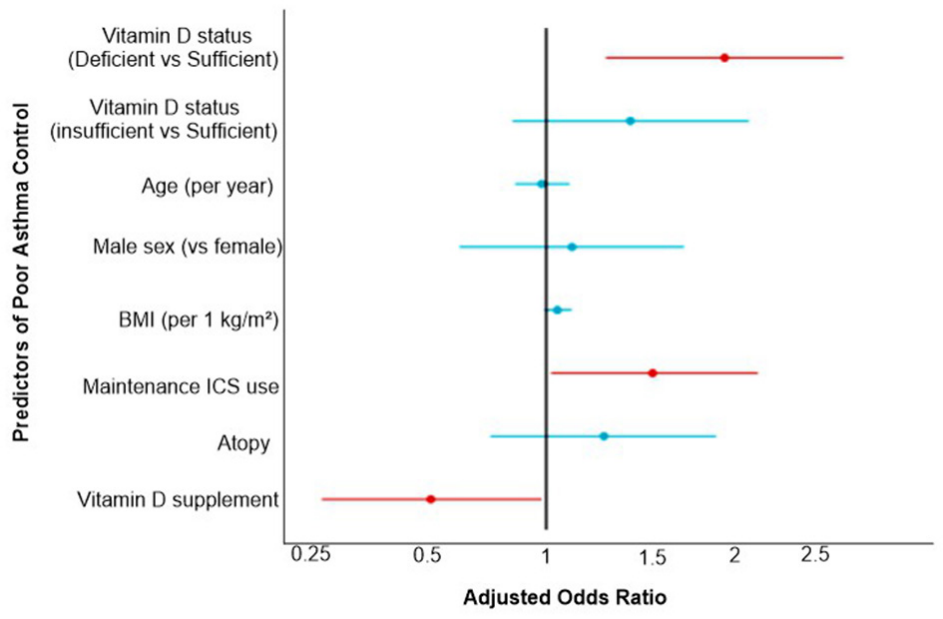
Multivariable logistic regression predicting poor asthma control.

The forest plot (Figure 4) shows that vitamin D deficiency significantly increased the odds of poor asthma control compared with sufficient levels (adjusted OR: 2.20, 95% CI: 1.30–3.72, *p* = 0.003), while insufficiency showed a non-significant trend. Use of maintenance inhaled corticosteroids was also associated with higher odds of poor control (OR: 1.60, *p* = 0.04), whereas vitamin D supplementation was protective (OR: 0.60, *p* = 0.042). Other predictors, including age, sex, BMI, and atopy, were not statistically significant.

### 3.6 Negative-binomial regression analysis

Negative-binomial regression analysis identified key predictors of asthma exacerbation count over the past 12 months. Vitamin D deficiency was associated with a 60% higher rate of exacerbations compared with sufficient children (Adjusted IRR: 1.60), whereas insufficient vitamin D showed no significant difference (IRR: 1.18). Maintenance ICS use increased exacerbation rate by 40% (IRR: 1.40). Use of vitamin D supplements was protective, reducing exacerbations by 28% (IRR: 0.72). Age, sex, BMI, and atopy were not significantly associated with exacerbation frequency (all *p* > 0.05).

### 3.7 Predictors of asthma control and exacerbation frequency in children

Analysis of logistic regression models indicated that children with vitamin D deficiency (<20 ng/ml) had significantly higher odds of poor asthma control (AOR: 2.38) and experiencing at least one exacerbation (AOR: 2.71) compared with those with sufficient levels. Reduced outdoor activity (<1 h/day) was associated with a 1.95-fold increased risk of poor asthma control (*p* = 0.026). Systemic corticosteroid uses and exposure to passive smoke were also significant predictors of having ≥1 exacerbation (AOR: 3.12 and 1.89, respectively). Negative-binomial regression analysis of exacerbation counts showed vitamin D deficiency and maintenance ICS use were linked to higher incidence rates (IRR: 1.60 and 1.40, respectively), whereas vitamin D supplementation demonstrated a protective effect (IRR: 0.72; *p* = 0.038).

**Figure 5:**
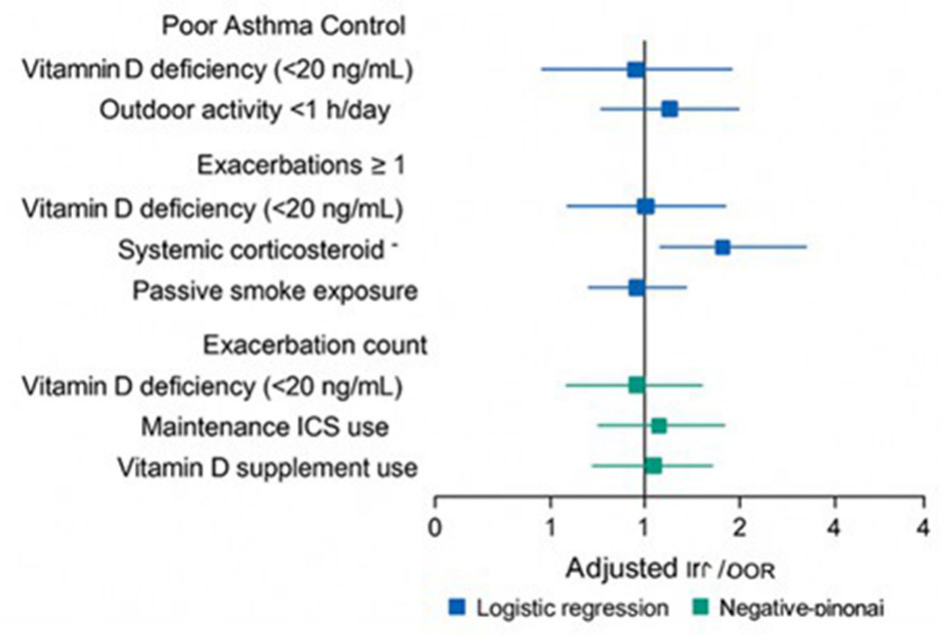
Key predictors of asthma control and exacerbation frequency in children.

Figure 5 explains the combined impact of logistic regression and negative binomial regression model for Key Predictors of Asthma Control and Exacerbation Frequency in Children. The regression models identified vitamin D deficiency as a consistent predictor of adverse asthma outcomes, with deficient children showing more than twofold higher odds of poor asthma control and increased risk of experiencing exacerbations compared with sufficient peers. Negative binomial analysis confirmed this association, indicating a 60% higher exacerbation rate in deficient children, while insufficient levels did not show a significant effect. Other factors such as low outdoor activity and corticosteroid use further amplified risk, whereas vitamin D supplementation was linked with fewer exacerbations, underscoring its potential protective role.

## 4 Discussion

The sociodemographic and lifestyle context that may contribute to both asthma control and vitamin D deficiency is enlisted in Table 1. Male predominance aligns with previous reports of higher asthma prevalence in boys during school age due to airway developmental differences (11). The relatively high proportion of overweight children suggests that obesity could be a contributing factor to poor asthma outcomes, consistent with recent meta-analyses linking obesity with asthma severity and treatment resistance. Importantly, the limited caregiver awareness and suboptimal adherence underscore gaps in community-level asthma education and self-management. Reduced outdoor activity, translating into low sunlight exposure, further explains the high prevalence of vitamin D deficiency (3). Together, these findings point to the need for integrated interventions targeting both lifestyle and public heal.

**Table 1:** Socio-demographic and clinical characteristics of children with asthma (*n* = 210).

**Variable**	**Category**	***N***	**%**	**Mean ±SD**
Age (years)	6–8	60	28.6	
	9–11	75	35.7	
	12–14	75	35.7	
Mean (age)				9.8 ± 2.5
Sex	Male	120	57.1	
	Female	90	42.9	
BMI (kg/m^2^)	Normal	140	66.7	18.5 ± 3.2
Residence	Urban	135	64.3	
	Rural	75	35.7	
Parental education	Primary or below	80	38.1	
	Secondary	65	31.0	
	Tertiary	65	31.0	
Family history of asthma	Yes	95	45.2	
	No	115	54.8	
Asthma duration (years)				4.1 ± 2.0
Vitamin D status (ng/ml)	Deficient (<12 ng/ml)	55	26.2	
	Insufficient (12–20 ng/ml)	80	38.1	
	Sufficient (20–30 ng/ml)	50	23.8	
	>30	25	11.9	
Asthma control	Well-controlled	110	52.4	19.5 ± 4.1
	Uncontrolled	100	47.6	18.2 ± 3.7
Exacerbations in past 12 months	0–1	100	47.6	
	2–3	70	33.3	
	≥4	40	19.0	

A biochemical and therapeutic profile that underscores the central role of vitamin D deficiency in asthma management is explained in Table 2. Despite nearly half of the children receiving supplementation, deficiency rates remained high, raising concerns about insufficient dosing or poor adherence (5, 8). This is consistent with evidence suggesting that supplementation strategies often fail to normalize serum vitamin D levels in high-risk pediatric populations (21). The reliance on rescue medications such as SABA and frequent systemic steroid use reflect suboptimal asthma control, reinforcing the clinical relevance of vitamin D status as a modifiable risk factor. Hospitalization rates further emphasize the health burden and resource utilization tied to poor control. When viewed alongside Table 1, these findings highlight that both socioeconomic determinants and biological insufficiencies interact to drive disease outcomes, suggesting a multidimensional approach is essential for effective public health interventions (21).

**Table 2:** Biochemical parameters and treatment profile of children with asthma (*n* = 210).

**Parameter**	***N***	**Mean ±SD/%**	**reference range**
Serum vitamin D (ng/ml)	210	19.2 ± 6.1	30–50
Serum calcium (mg/dl)	210	8.7 ± 0.9	8.5–10.5
Vitamin D supplementation	98	46.7%	
Inhaled corticosteroid (ICS) use	120	57.1%	
Short-acting β_2_-agonist (SABA) use	165	78.6%	
Systemic steroid use (past 12 months)	48	22.9%	
Hospitalization (past 12 months)	48	22.9%	

The biochemical profiles outlined in Supplementary Table S4 provide strong evidence that vitamin D deficiency represents a key biological substrate influencing asthma outcomes in this cohort (18). Nearly 69% of children exhibited serum vitamin D concentrations below the recommended reference range, with a mean level of 19.2 ± 6.1 ng/ml, placing the majority in the deficient category. This aligns with findings in recent pediatric asthma studies, where vitamin D insufficiency has been repeatedly associated with greater airway hyperreactivity, impaired lung function, and heightened risk of exacerbations (5).

The regression analyses reinforce these biochemical trends by showing that vitamin D deficiency was an independent predictor of poor asthma control, frequent exacerbations, and increased hospitalization rates (8). The multivariable logistic regression model revealed that children with deficiency had significantly higher odds of uncontrolled asthma compared to their sufficient counterparts, even after adjusting for age, sex, and BMI. These results suggest that low vitamin D is not merely a background nutritional issue but actively contributes to the clinical trajectory of asthma (22, 23).

The interplay between vitamin D and calcium metabolism further supports this interpretation. While most calcium and phosphate levels were within normal ranges, subgroups showed hypocalcemia and altered phosphate balance, likely reflecting secondary consequences of chronic vitamin D deficiency (17, 24). Such imbalances may exacerbate airway inflammation and immune dysregulation, creating a biochemical environment conducive to recurrent asthma symptoms (16). The presence of elevated parathyroid hormone (6.7%) in some children also suggests compensatory mechanisms, which have been linked to poor respiratory outcomes when persistent (25).

The treatment and clinical profile of the cohort (Supplementary Table S5) highlights important patterns in asthma management and disease burden. More than half of the children (58.1%) were regular users of inhaled corticosteroids (ICS), indicating adherence to guideline-based preventive therapy in a substantial proportion, yet nearly one in five still required oral steroid bursts within the past year, reflecting episodes of poor control or acute exacerbations (26). Use of leukotriene receptor antagonists was reported in 30.5% of participants, suggesting their role as an add-on therapy in children with persistent symptoms. Despite the relatively high rate of controller medication use, 17.1% of children experienced hospitalizations and 24.8% required at least one emergency room visit in the last year, underscoring gaps in asthma control and ongoing morbidity. These findings indicate that while pharmacological management is common, a significant proportion of children remain at risk of severe exacerbations, highlighting the need for integrated approaches that address treatment adherence, environmental triggers, and modifiable risk factors such as vitamin D deficiency (26).

By integrating these biochemical abnormalities with regression outcomes, the study underscores a clinically relevant pathway where vitamin D deficiency contributes to asthma morbidity through impaired mineral metabolism, immune dysregulation, and secondary hormonal effects (27). The findings justify targeted interventions such as routine biochemical screening and vitamin D supplementation programs in children with asthma, which could reduce exacerbation frequency and improve long-term control (22, 28). From a public health perspective, these results demonstrate the importance of nutritional determinants of respiratory disease, supporting the broader goal of preventive strategies within community health frameworks (22).

Table 3 highlight the findings suggest a strong gradient linking lower vitamin D levels with worse asthma outcomes. Children with deficiency were more likely to experience poor control, frequent exacerbations, and higher hospitalization rates. This pattern aligns with prior evidence indicating that vitamin D plays a protective role in airway inflammation and immune modulation, which may reduce susceptibility to asthma exacerbations and improve control (17). The observed dose–response trend across categories supports the hypothesis that even modest improvements in vitamin D levels may have clinical relevance. Similar associations have been reported in mechanistic studies, where vitamin D deficiency amplifies airway hyperresponsiveness and increases infection risk, thereby worsening asthma outcomes (29). Although causality cannot be inferred from this retrospective analysis, the consistency of associations highlights the potential value of vitamin D optimization as a complementary strategy in pediatric asthma management.

**Table 3:** Unadjusted comparisons for key outcomes (selected).

**Outcome**	**Deficient (*n* = 95)**	**Insufficient (*n* = 65)**	**Sufficient (*n* = 50)**	***P*-value**
Proportion poorly controlled asthma	60/95 (63.2%)	28/65 (43.1%)	12/50 (24.0%)	0.001
Mean exacerbations (12 months)	2.6 ± 1.3	1.9 ± 1.1	1.1 ± 0.9	0.001
Hospitalization rate (any)	30/95 (31.6%)	12/65 (18.5%)	6/50 (12.0%)	0.014

The retrospective nature of this study limited our sample to children with a confirmed diagnosis of asthma; therefore, a healthy control group was not available for direct comparison. While an association between low vitamin D levels and poor asthma control was observed, it is not possible to attribute poor control exclusively to vitamin D deficiency due to potential confounding factors. Notably, variability in prescribed medication types, dosages, and adherence among participants may have influenced asthma control independently of vitamin D status. These factors contributed to heterogeneity in outcomes and complicate the interpretation of any direct relationship between vitamin D levels and asthma morbidity.

The sensitivity analysis (Supplementary Table S6) shows a clear gradient: children with severe vitamin D deficiency (<12 ng/ml) had the highest rates of poor asthma control, exacerbations, emergency visits, and hospitalizations, while those with sufficient levels (>30 ng/ml) had the lowest (21). The persistence of this dose–response relationship across alternative cutoffs confirms the robustness of the main findings.

These adjusted models demonstrate that the observed associations between vitamin D deficiency and asthma outcomes are not explained by demographic or socioeconomic factors. The increased odds of poor asthma control and exacerbations in deficient children are consistent with mechanistic studies showing vitamin D regulates T-cell activation and airway inflammation. The findings also align with pediatric clinical studies reporting reduced exacerbation risk after vitamin D supplementation (30). The independent contribution of low socioeconomic status suggests that environmental and behavioral determinants interact with biological pathways, reinforcing the importance of addressing both nutritional deficiencies and social inequalities in asthma prevention (31). From a public health perspective, these results support integrated interventions, combining vitamin D monitoring with broader strategies to reduce pediatric asthma burden.

**Figure 6:**
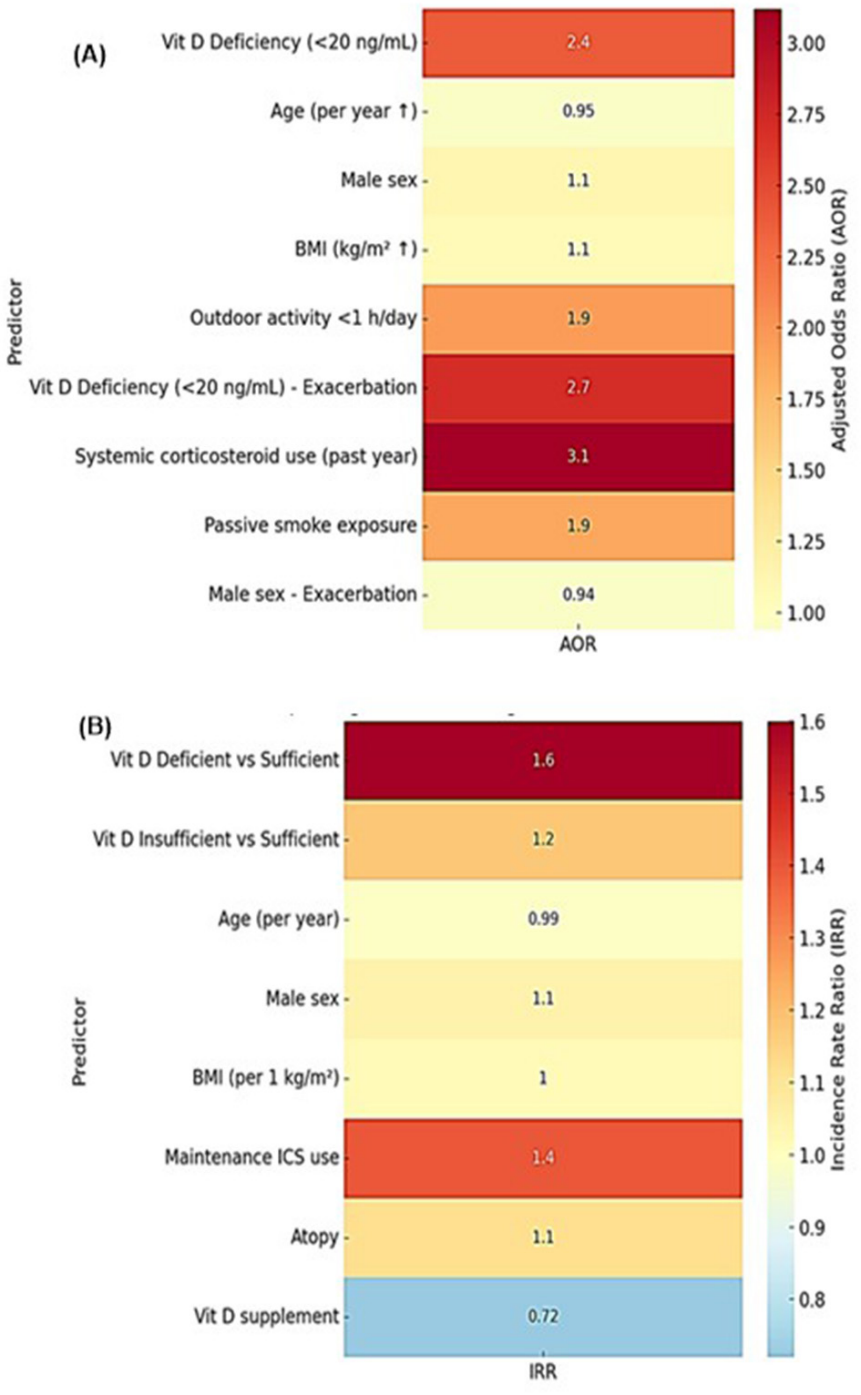
**(A)** Heat map of logistic regression predictors for asthma outcomes, **(B)** Heat map of negative binomial regression predictors for exacerbation count.

The analyses of asthma outcomes in school-aged children highlight vitamin D deficiency as a consistent and independent predictor of both poor asthma control and higher exacerbation risk (8, 32). Univariate logistic regression (Table 4) indicated that vitamin D deficiency (<20 ng/ml) was associated with increased odds of poor asthma control and ≥1 exacerbation, while other demographic factors, including age, sex, and BMI, were not significant (3). The heatmap (Figure 6A) highlights that vitamin D deficiency and reduced outdoor activity are strongly associated with poor asthma control, while vitamin D deficiency, systemic corticosteroid use, and passive smoke exposure are key predictors of exacerbations (33).

**Table 4:** Logistic regression analysis of vitamin D deficiency and asthma outcomes in children (*n* = 210).

**Outcome variable**	**Predictor**	**Adjusted odds ratio (AOR)**	**95% confidence interval (CI)**	***P*-value**
Poor asthma control	Vitamin D deficiency (<20 ng/ml)	2.38	1.34–4.22	0.003
	Age (per year ↑)	0.95	0.87–1.05	0.310
	Male sex	1.12	0.63–1.99	0.701
	BMI (kg/m^2^ ↑)	1.08	0.99–1.19	0.072
	Outdoor activity <1 h/day	1.95	1.08–3.51	0.026
Asthma exacerbations (≥1 in past year)	Vitamin D deficiency (<20 ng/ml)	2.71	1.41–5.19	0.003
	Systemic corticosteroid use (past year)	3.12	1.41–6.87	0.005
	Exposure to passive smoke	1.89	1.01–3.53	0.045
	Male sex	0.94	0.50–1.78	0.850

Darker color intensity indicates higher adjusted odds ratios, making it easy to visualize the relative strength of each risk factor (25, 27, 28). Multivariable logistic regression models (Table 5) confirmed these associations, showing that vitamin D deficiency remained a strong predictor of poor control (adjusted OR: 2.20; 95% CI: 1.30–3.72; *p* = 0.003) and ≥1 exacerbation (adjusted OR: 2.71; 95% CI: 1.41–5.19; *p* = 0.003), whereas insufficient vitamin D levels did not reach statistical significance (OR: 1.45; *p* = 0.16), suggesting a threshold effect. Vitamin D supplementation reduced the odds of poor asthma control (OR: 0.60; 95% CI: 0.37–0.98; *p* = 0.042) and lowered exacerbation incidence (IRR: 0.72; *p* = 0.038), emphasizing its potential as a preventive strategy (25).

**Table 5:** Multivariable logistic regression predictors of poor asthma control (binary outcome).

**Predictor**	**Adjusted OR**	**95% CI**	***P*-value**
Vitamin D status (deficient vs. sufficient)	2.20	1.30–3.72	0.003
Vitamin D status (insufficient vs. sufficient)	1.45	0.86–2.45	0.16
Age (per year)	0.98	0.87–1.11	0.75
Male sex (vs. female)	1.12	0.68–1.84	0.65
BMI (per 1 kg/m^2^)	1.05	0.99–1.12	0.10
Maintenance ICS use (yes)	1.60	1.02–2.55	0.04
Atopy (yes)	1.29	0.78–2.12	0.33
Vitamin D supplement (yes)	0.60	0.37–0.98	0.042

Negative-binomial regression analysis of exacerbation counts (Table 6) further supported these findings, showing higher incidence rates in vitamin D–deficient children (IRR: 1.60; 95% CI: 1.20–2.15; *p* = 0.002). Maintenance inhaled corticosteroid (ICS) use was associated with both higher odds of poor control (OR: 1.60; 95% CI: 1.02–2.55; *p* = 0.04) and higher exacerbation counts (IRR: 1.40; *p* = 0.02), likely reflecting those children on ICS represent a more severe disease subgroup rather than a causal effect of therapy (28). Systemic corticosteroid uses also increased exacerbation risk (OR: 3.12; 95% CI: 1.41–6.87; *p* = 0.005), consistent with frequent steroid courses indicating unstable asthma (27). The heatmap (Figure 6B) shows that vitamin D deficiency and maintenance ICS use are linked with higher exacerbation rates, whereas vitamin D supplementation appears protective. Stronger color intensity reflects higher incidence rate ratios, helping to distinguish significant predictors from non-significant ones (8).

**Table 6:** Negative-binomial regression predictors of exacerbation count (12 months).

**Predictor**	**Adjusted IRR**	**95% CI**	***P*-value**
Vitamin D status (deficient vs. sufficient)	1.60	1.20–2.15	0.002
Vitamin D status (insufficient vs. sufficient)	1.18	0.89–1.56	0.25
Age (per year)	0.99	0.92–1.07	0.80
Male sex (vs. female)	1.05	0.86–1.28	0.64
BMI (per 1 kg/m^2^)	1.02	0.99–1.05	0.25
Maintenance ICS use (yes)	1.40	1.05–1.87	0.02
Atopy (yes)	1.12	0.86–1.47	0.40
Vitamin D supplement (yes)	0.72	0.53–0.98	0.038

IRR, incidence rate ratio.

Interpretation: IRR >1 indicates higher exacerbation rate.

Environmental and behavioral factors significantly influenced outcomes. Reduced outdoor activity (<1 h/day) was linked to poor asthma control (OR: 1.95; 95% CI: 1.08–3.51; *p* = 0.026), likely reflecting limited sun exposure contributing to vitamin D deficiency (15, 23). Passive smoke exposure independently increased the likelihood of ≥1 exacerbation (OR: 1.89; 95% CI: 1.01–3.53; *p* = 0.045), confirming the role of modifiable environmental exposures. Other demographic and clinical factors, including age, sex, BMI, and atopy, were not independently associated with outcomes, indicating that modifiable nutritional and environmental factors have a greater impact on short-term asthma morbidity (22, 27).

Finally, the summary of key predictors in Table 7 and the visualization in Figure 5 emphasize that vitamin D deficiency, supplementation, outdoor activity, ICS use, and passive smoke exposure collectively influence asthma control and exacerbation burden. These findings support a multifactorial approach in pediatric asthma management, integrating vitamin D assessment, supplementation, environmental interventions, and promotion of healthy outdoor activity to optimize control and reduce exacerbations (15, 17, 22, 24, 27).

**Table 7:** Key predictors of asthma control and exacerbation frequency in children (*n* = 210).

**Outcome**	**Predictor**	**Model**	**Adjusted effect**	**95% CI**	***P*-value**	**Interpretation**
Poor asthma control	Vitamin D deficiency (<20 ng/ml)	Logistic regression	AOR: 2.38	1.34–4.22	0.003	Higher odds of poor control
Poor asthma control	Outdoor activity <1 h/day	Logistic regression	AOR: 1.95	1.08–3.51	0.026	Increased risk
Exacerbations ≥1	Vitamin D deficiency (<20 ng/ml)	Logistic regression	AOR: 2.71	1.41–5.19	0.003	Higher odds of ≥1 exacerbation
Exacerbations ≥1	Systemic corticosteroid use	Logistic regression	AOR: 3.12	1.41–6.87	0.005	Higher risk
Exacerbations ≥1	Passive smoke exposure	Logistic regression	AOR: 1.89	1.01–3.53	0.045	Increased risk
Exacerbation count	Vitamin D deficiency (<20 ng/ml)	Negative-binomial	IRR: 1.60	1.20–2.15	0.002	Higher incidence rate
Exacerbation count	Maintenance ICS use	Negative-binomial	IRR: 1.40	1.05–1.87	0.02	Increased incidence
Exacerbation count	Vitamin D supplement use	Negative-binomial	IRR: 0.72	0.53–0.98	0.038	Protective effect

## 5 Limitations and future perspective

This study's retrospective design restricted our analysis to data available in medical records, potentially introducing selection bias and constraining our ability to control for confounding variables. Heterogeneous treatment patterns, including differences in medication regimens and dosages, further limited the ability to isolate the impact of vitamin D deficiency on asthma morbidity. Future research should employ standardized protocols for asthma diagnosis and management, and consider active involvement of patients and caregivers in selecting treatment strategies and hospitalization criteria to enhance data consistency and relevance. The retrospective design may introduce selection bias and limit the generalizability of findings, although internal consistency across clinical variables supports reliability of the observed associations.

## 6 Conclusion

This retrospective cohort study of school-age children with asthma demonstrates a clear association between vitamin D deficiency and adverse clinical outcomes, including poor asthma control, higher exacerbation frequency, and increased hospitalization rates. The majority of children were found to have suboptimal serum vitamin D concentrations, which correlated with both questionnaire-based assessments and objective biochemical markers. Regression analyses confirmed that deficiency remained an independent predictor of morbidity even after accounting for demographic and treatment factors. These findings highlight the importance of nutritional determinants in pediatric asthma and provide new evidence that vitamin D status is a modifiable risk factor.

From a public health perspective, the results emphasize the need for screening programs and preventive strategies targeting vitamin D deficiency in children with asthma. Integrating biochemical monitoring with tailored supplementation could reduce the disease burden, improve quality of life, and lower healthcare utilization. By connecting individual-level biological markers to population-level outcomes, this study contributes to the growing recognition that nutritional interventions should form a core component of asthma management and prevention policies.

## Data Availability

The original contributions presented in the study are included in the article/Supplementary material, further inquiries can be directed to the corresponding author.

## References

[1] Zhu Y Jing D Liang H Li D Chang Q Shen M . Vitamin D status and asthma, lung function, and hospitalization among British adults. Front Nutr. (2022) 9:954768. doi: 10.3389/fnut.2022.954768 36034921 PMC9399919

[2] Alkhatatbeh MJ Almomani HS Abdul-Razzak KK Samrah S. Association of asthma with low serum vitamin D and its related musculoskeletal and psychological symptoms in adults: a case-control study. NPJ Prim Care Respir Med. (2021) 31:27. doi: 10.1038/s41533-021-00239-7 33990605 PMC8121852

[3] Bakdounes D Dughly R Almasri I Martini N Hanna M Albelal D . High prevalence of uncontrolled asthma and its association with obesity and GERD-related symptoms in Syria: a multicenter cross-sectional study. Health Sci Rep. (2025) 8:e70828. doi: 10.1002/hsr2.70828 40406649 PMC12095846

[4] Alolayan A Al-Wutayd O. Association between vitamin D status and asthma control levels among children and adolescents: a retrospective cross sectional study in Saudi Arabia. BMC Pediatr. (2025) 25:585. doi: 10.1186/s12887-025-05969-y 40751233 PMC12315271

[5] Ogeyingbo OD Ahmed R Gyawali M Venkatesan N Bhandari R Botleroo RA . The relationship between vitamin D and asthma exacerbation. Cureus. (2021) 13. doi: 10.7759/cureus.17279 34462708 PMC8389855

[6] Shen Q. Protection against cigarette smoke-induced chronic obstructive pulmonary disease via activation of the SIRT1/FoxO1 axis by targeting microRNA-132. Am J Transl Res. (2024) 16:5516–24. doi: 10.62347/FVQP4019 39544778 PMC11558385

[7] Hao M Xu R Luo N Liu M Xie J Zhang W. The effect of vitamin d supplementation in children with asthma: a meta-analysis. Front Pediatr. (2022) 10:840617. doi: 10.3389/fped.2022.840617 35844729 PMC9277022

[8] Fedora K Setyoningrum RA Aina Q Rosyidah LN Ni'mah NL Titiharja FF. Vitamin D supplementation decrease asthma exacerbations in children: a systematic review and meta-analysis of randomized controlled trials. Ann Med. (2024) 56:2400313. doi: 10.1080/07853890.2024.2400313 39421966 PMC11492411

[9] Bin Y Peng R Lee Y Lee Z Liu Y. Efficacy of Xuebijing injection on pulmonary ventilation improvement in acute pancreatitis: a systematic review and meta-analysis. Front Pharmacol. (2025) 16:1549419. doi: 10.3389/fphar.2025.1549419 40308770 PMC12041077

[10] Li Q Zhou Q Zhang G Tian X Li Y Wang Z . Vitamin D supplementation and allergic diseases during childhood: a systematic review and meta-analysis. Nutrients. (2022) 14:3947. doi: 10.3390/nu14193947 36235600 PMC9571357

[11] Liu B Du H Zhang J Jiang J Zhang X He F . Developing a new sepsis screening tool based on lymphocyte count, international normalized ratio and procalcitonin (LIP score). Sci Rep. (2022) 12:20002. doi: 10.1038/s41598-022-16744-9 36411279 PMC9678875

[12] Global Initiative for Asthma. Global Strategy for Asthma Management and Prevention. Global Initiative for Asthma (2024). Available online at: https://ginasthma.org (Accessed January 15, 2025).

[13] Li J Liang L Lyu B Cai YS Zuo Y Su J. et al. Double trouble: the interaction of PM25 and O3 on respiratory hospital admissions. Environ Pollut. (2023) 338:122665. doi: 10.1016/j.envpol.2023.122665 37806428

[14] Lizzo JM Goldin J Cortes S. Pediatric Asthma. Treasure Island, FL: StatPearls Publishing (2025). 31869095

[15] Raymond-Lezman JR Riskin SI. Benefits and risks of sun exposure to maintain adequate vitamin D levels. Cureus. (2023) 15. doi: 10.7759/cureus.38578 37284402 PMC10239563

[16] Krasowski R Kamińska K Głodek K Ostrowska J Zajda K Pawliczak R . The therapeutic potential of vitamin D supplementation in asthma. Pharmacol Rep. (2025) 77:874–88. doi: 10.1007/s43440-025-00734-5 40392518 PMC12241240

[17] Salmanpour F Kian N Samieefar N Khazeei Tabari MA Rezaei N. Asthma and vitamin D deficiency: occurrence, immune mechanisms, and new perspectives. J Immunol Res. (2022) 2022:1–7. doi: 10.1155/2022/6735900 35874901 PMC9307373

[18] Wang Y Lao W Guo S. Impact of vitamin D supplementation on clinical outcomes in children with asthma. Int J Pharmacol. (2023) 19:714–21. doi: 10.3923/ijp.2023.714.721

[19] Yang S Zhang S Cao Q Zhu G Liu J Li G . Association between oral microbial diversity (only bacteria) and diabetes in U.S. adults: analysis of NHANES 2009–2012 data. BMC Oral Health. (2025) 25:837. doi: 10.1186/s12903-025-06204-x 40437450 PMC12121004

[20] Peña-Durán E García-Galindo JJ López-Murillo LD Huerta-Huerta A Balleza-Alejandri LR Beltrán-Ramírez A . Microbiota and inflammatory markers: a review of their interplay, clinical implications, and metabolic disorders. Int J Mol Sci. (2025) 26:1773. doi: 10.3390/ijms26041773 40004236 PMC11854938

[21] Martineau AR Jolliffe DA Hooper RL Greenberg L Aloia JF Bergman P . Vitamin D supplementation to prevent acute respiratory tract infections: systematic review and meta-analysis of individual participant data. BMJ. (2017) 56:i6583. doi: 10.1136/bmj.i6583 28202713 PMC5310969

[22] Lei Y Luo Y Wang Y Liu C Luo L Li J. The association between vitamin D intake and the prevalence and mortality of asthma in the US adults. Nutr J. (2025) 24:103. doi: 10.1186/s12937-025-01171-z 40605091 PMC12217372

[23] Asseri A. Serum vitamin D profiles of children with asthma in southwest Saudi: a comparative cross-sectional study. Int J Gen Med. (2024) 17:6323–33. doi: 10.2147/IJGM.S503293 39717072 PMC11663989

[24] Zhou L Han C Zhou Y. The role of severe vitamin D deficiency in predicting the risk of severe exacerbation in patients with chronic obstructive pulmonary disease. Int J Chron Obstruct Pulmon Dis. (2025) 20:171–9. doi: 10.2147/COPD.S489650 39867990 PMC11766290

[25] Sobczak M Pawliczak R. Relationship between vitamin D and asthma from gestational to adulthood period: a meta-analysis of randomized clinical trials. BMC Pulm Med. (2023) 23:212. doi: 10.1186/s12890-023-02514-4 37330474 PMC10276459

[26] Tibrewal C Modi NS Bajoria PS Dave PA Rohit RK Patel P . Therapeutic potential of vitamin D in management of asthma: a literature review. Cureus. (2023) 20:171–9. doi: 10.7759/cureus.41956 37588324 PMC10425698

[27] Hamad Khan M. Role of inhaled corticosteroids versus systemic corticosteroids in the management of asthma. J Popul Ther Clin Pharmacol. (2024) 23:1384–92. doi: 10.53555/8kky7827

[28] Averell CM Laliberté F Germain G Duh MS Rousculp MD MacKnight SD . Impact of adherence to treatment with inhaled corticosteroids/long-acting β-agonists on asthma outcomes in the United States. Ther Adv Respir Dis. (2022) 16:41956. doi: 10.1177/17534666221116997 36036456 PMC9434680

[29] Fares MM Alkhaled LH Mroueh SM Akl EA. Vitamin D supplementation in children with asthma: a systematic review and meta-analysis. BMC Res Notes. (2015) 8:23. doi: 10.1186/s13104-014-0961-3 25643669 PMC4328422

[30] Wu Y Zeng M Jiao X Ma X Wang H Chen Y . Protective effects of pollenin B in asthma: PPAR-γ-mediated regulation of inflammatory pathways and arachidonic acid metabolism. Phytomedicine. (2025) 145:156975. doi: 10.1016/j.phymed.2025.156975 40544731

[31] Vieth R. Vitamin D supplementation: cholecalciferol, calcifediol, and calcitriol. Eur J Clin Nutr. (2020) 16. doi: 10.1038/s41430-020-0697-1 32704098

[32] Liu M Wang J Sun X. A meta-analysis on vitamin D supplementation and asthma treatment. Front Nutr. (2022) 9:23. doi: 10.3389/fnut.2022.860628 35873428 PMC9300755

[33] Williamson A Martineau AR Sheikh A Jolliffe D Griffiths CJ. Vitamin D for the management of asthma. Cochrane Database Syst Rev. (2023) 2023:156975. doi: 10.1002/14651858.CD011511.pub3 36744416 PMC9899558

