# Supplementary material for: Vitamin D deficiency and asthma morbidity in school-age children: a single-center cohort study
Source: Front Public Health. 2025 Dec 15;13:1717912. doi: 10.3389/fpubh.2025.1717912 (PMC12750622; doi:10.3389/fpubh.2025.1717912)
Supplement: Supplementary file 1 [file Table_1.docx]

**Vitamin D Deficiency and Asthma Morbidity in School-Age Children: A Single-Centre Cohort Study**

**Supplementary**

## Table S1. Public Health Questionnaire for Assessing Vitamin D Status and Asthma Outcomes in School-Age Children (n = 210)

| **Section** | **Question / Item** | **Response Options** | **Coding / Notes** |
| --- | --- | --- | --- |
| **Demographics** | Age (years) | Integer | Use recorded birthdate, calculate to nearest year |
|  | Sex | Male / Female / Other | 1 = Male, 2 = Female, 3 = Other |
|  | Residence | Urban / Suburban / Rural | 1 / 2 / 3 |
|  | Parental Education | None / Primary / Secondary / College / University | Ordinal |
| **Vitamin D / Exposure** | Sun exposure (daily) | <15 min / 15–30 / 30–60 / >60 | 1 / 2 / 3 / 4 |
|  | Use of sunblock | Yes / No | 1 / 0 |
|  | Diet rich in vitamin D (fish, fortified milk) | Yes / No / Unknown | 1 / 0 / 9 |
|  | Current vitamin D supplement use | Yes / No | 1 / 0 |
|  | Supplement daily dose (IU or mcg) | Numeric | If yes above |
| **Asthma history & control** | Age at asthma diagnosis | Integer (years) |  |
|  | Duration of asthma (years) | Integer |  |
|  | Exacerbations (past 12 months) | Integer | Count of events requiring systemic steroids / hospital / ED |
|  | ED visits for asthma (past 12 months) | Yes / No / Number | 1 / 0 + integer |
|  | Hospitalizations for asthma (past 12 mo) | Yes / No / Number | 1 / 0 + integer |
|  | Unscheduled clinic visits (past 12 mo) | Integer |  |
|  | Days missed school due to asthma | Integer |  |
| **Asthma Control (ACT / C-ACT)** | (Insert validated item set) | e.g., 5-item scale (1–5) for ACT, 7-item for C-ACT | Sum or mean score; indicate controlled or not based on cut-off |
| **Medication & Treatment** | Maintenance inhaled corticosteroid use | Yes / No | 1 / 0 |
|  | Use of leukotriene receptor antagonist | Yes / No | 1 / 0 |
|  | Use of long-term oral steroids | Yes / No | 1 / 0 (if yes, likely exclusion) |
| **Other factors / comorbidities** | Atopy / allergic rhinitis / eczema | Yes / No / Unknown | 1 / 0 / 9 |
|  | Passive smoking exposure | Yes / No | 1 / 0 |
|  | Indoor air pollution (biomass fuel etc) | Yes / No | 1 / 0 |

## Table S2. Scoring Framework for Public Health Questionnaire

| **Domain** | **Item** | **Response Options** | **Score Coding** | **Composite Score & Categories** |
| --- | --- | --- | --- | --- |
| **Sunlight Exposure Score (SSES)** | Outdoor playtime per day | <30 min = 0; 30–60 min = 1; >60 min = 2 | 0–2 | **SSES = sum of playtime + clothing + sunblock**  Range: 0–5Low: 0–1; Moderate: 2–3; High: 4–5 |
|  | Clothing outdoors | Covered = 0; Partial = 1; Uncovered = 2 | 0–2 |  |
|  | Regular sunblock use | Yes = 0; No = 1 | 0–1 |  |
| **Nutrition & Supplementation Score (NSS)** | Daily milk intake | None = 0; 1 cup = 1; 2+ cups = 2 | 0–2 | **NSS = milk + fish/eggs + supplements**  Range: 0–6Poor: 0–2; Adequate: 3–4; High: 5–6 |
|  | Oily fish/eggs | Rarely = 0; Weekly = 1; ≥3 times weekly = 2 | 0–2 |  |
|  | Vitamin D supplements | No = 0; <400 IU/day = 1; ≥400 IU/day = 2 | 0–2 |  |
| **Environmental Risk Score (ERS)** | Passive smoking at home | Yes = 1; No = 0 | 0–1 | **ERS = smoke + biomass + crowding** Range: 0–3Low = 0; Moderate = 1; High = 2–3 |
|  | Biomass fuel use | Yes = 1; No = 0 | 0–1 |  |
|  | Crowding (≥4 persons/room) | Yes = 1; No = 0 | 0–1 |  |
| **Healthcare Access & Adherence Score (HAAS)** | Regular follow-up visits | Yes = 1; No = 0 | 0–1 | **HAAS = follow-up + controller + adherence**  Range: 0–3Poor = 0; Partial = 1–2; Good = 3 |
|  | Controller medication use | Yes = 1; No = 0 | 0–1 |  |
|  | Reported adherence | Good = 1; Poor = 0 | 0–1 |  |
| **Asthma Outcomes** | Exacerbations in past 12 months | Numeric count | Mean ± SD | Modeled with Poisson/negative binomial regression |
|  | Hospitalizations (past year) | Yes = 1; No = 0 | Binary | Logistic regression |
|  | Asthma control (ACT/C-ACT) | Controlled = 1; Poor control = 0 | Binary | Logistic regression / adjusted OR |
|  | Severity index (optional) | Z-score composite: exacerbations + control + hospitalizations | Continuous | Linear regression |

**Table S3. Socio-demographic Factors Associated with Vitamin D Deficiency**

| **Variable** | **Deficient (n = 144)** | **Sufficient (n = 66)** | **p-value** |
| --- | --- | --- | --- |
| Age (years, Mean ± SD) | 10.1 ± 2.8 | 10.6 ± 2.5 | 0.162 |
| Male sex, n (%) | 92 (63.9) | 37 (56.0) | 0.311 |
| BMI (kg/m², Mean ± SD) | 18.2 ± 3.1 | 19.0 ± 2.9 | 0.094 |
| Outdoor activity < 1 h/day, n (%) | 108 (75.0) | 28 (42.4) | <0.001 |
| Sunscreen use, n (%) | 39 (27.1) | 9 (13.6) | 0.047 |

**Table S4. Biochemical Assays and Reference Ranges**

| **Parameter** | **Mean ± SD** | **Reference Range** | **% Below Reference** | **% Above Reference** |
| --- | --- | --- | --- | --- |
| Serum Vitamin D (ng/mL) | 19.2 ± 6.1 | 30–50 | 68.6% | 2.4% |
| Serum Calcium (mg/dL) | 8.7 ± 0.9 | 8.5–10.5 | 12.9% | 1.9% |
| Serum Phosphate (mg/dL) | 3.6 ± 0.6 | 2.5–4.5 | 7.6% | 3.3% |
| Parathyroid Hormone (pg/mL) | 48.2 ± 12.5 | 10–65 | 4.8% | 6.7% |

**Table S5. Treatment and Clinical Profile**

| **Variable** | **N** | **%** |
| --- | --- | --- |
| Regular ICS users | 122 | 58.1% |
| Leukotriene antagonist users | 64 | 30.5% |
| Oral steroid bursts (≥1 in last year) | 41 | 19.5% |
| Hospitalizations due to asthma (last year) | 36 | 17.1% |
| Emergency room visits (≥1 in last year) | 52 | 24.8% |

### **Table S6. Sensitivity Analysis of Asthma Morbidity Outcomes Using Alternative Vitamin D Cutoffs**

| **Vitamin D Category (ng/mL)** | **n** | **(%)** | **Poor Asthma Control (%)** | **≥1 Exacerbation in Past Year (%)** | **Emergency Visits (%)** | **Hospitalization (%)** |
| --- | --- | --- | --- | --- | --- | --- |
| **<12 (Severe Deficiency)** | 42 | 20 | 61.9 | 54.8 | 40.5 | 28.6 |
| **12–20 (Deficiency)** | 53 | 25 | 49.1 | 43.4 | 30.2 | 20.8 |
| **20–30 (Insufficiency)** | 67 | 31 | 34.3 | 28.4 | 17.9 | 11.9 |
| **>30 (Sufficiency)** | 48 | 22 | 20.8 | 16.7 | 10.4 | 6.3 |
